# Liver injury during durvalumab-based immunotherapy is associated with poorer patient survival: A retrospective analysis

**DOI:** 10.3389/fonc.2022.984940

**Published:** 2022-10-24

**Authors:** Linnea A. Swanson, Ihab Kassab, Irene Tsung, Bryan J. Schneider, Robert J. Fontana

**Affiliations:** ^1^ Department of Internal Medicine, University of Michigan, Ann Arbor, MI, United States; ^2^ Division of Hematology and Oncology, University of Michigan, Ann Arbor, MI, United States; ^3^ Division of Gastroenterology and Hepatology, University of Michigan, Ann Arbor, MI, United States

**Keywords:** checkpoint inhibitors, immunotherapy, drug hepatotoxicity, immune-mediated liver injury, drug induced liver injury, Roussel Uclaf Causality Assessment Method, biliary tract cancer, lung cancer

## Abstract

**Background:**

Durvalumab is approved for the treatment of lung cancer, advanced biliary tract cancers, and is also being evaluated in many other solid organ tumors. The aim of our study is to define the incidence, etiology, and outcomes of liver injury in consecutive patients receiving durvalumab-based immunotherapy.

**Patients and methods:**

Durvalumab treated patients between 1/2016 – 7/2020 were identified from the electronic medical record. Liver injury was defined as serum AST or ALT ≥ 5x upper limit of normal (ULN), ALP ≥ 2x ULN, bilirubin ≥ 2.5 mg/dl, or INR ≥ 1.5. Potential drug induced liver injury (DILI) cases were adjudicated using expert opinion scoring and confirmed with Roussel Uclaf Causality Assessment Method (RUCAM).

**Results:**

Amongst 112 patients, 58 (52%) had non-small cell lung cancer, the median age was 65 years, and 60% were male. The 21 (19%) liver injury patients were significantly more likely to harbor hepatic metastases (52% vs 17%, p=<0.001), experience tumor progression (67% vs 32%, p=0.01) or die (48% vs 11%, p<0.001) during follow-up compared to the 91 without liver injury. Using multivariate regression analysis, the development of liver injury during treatment as well as baseline hepatic metastases were independently associated with mortality during follow-up. Six of the 21 (29%) liver injury cases were adjudicated as probable DILI with four attributed to durvalumab and two due to other drugs (paclitaxel, pembrolizumab). Durvalumab was permanently discontinued in two DILI patients, three received corticosteroids, and one was successfully rechallenged. Only one patient with DILI developed jaundice, and none required hospitalization. Liver biochemistries normalized in all 6 DILI cases, while they only normalized in 27% of the 15 non-DILI cases (p=0.002). The 6 DILI patients also had a trend towards improved survival compared to those with other causes of liver injury

**Conclusion:**

Liver injury was observed in 19% of durvalumab-treated patients and is associated with a greater likelihood of tumor progression and death during follow-up. The four durvalumab DILI cases were mild and self-limited, highlighting the importance of causality assessment to determine the cause of liver injury in oncology patients receiving immunotherapy.

## Introduction

Durvalumab is an immune checkpoint inhibitor (ICI) that selectively binds to Programmed cell-death 1 ligand 1 (PD-L1) and inhibits its interactions with Programmed cell death-1 (PD-1) and CD80 resulting in T cell activation and anti-tumor responses (1). Durvalumab is currently approved as a single agent for non-small cell lung cancer (NSCLC), in combination with other agents for small cell lung cancer (SCLC) and most recently in 2022, gained approval for advanced biliary tract cancer in combination with chemotherapy (2, 3). Notably, durvalumab is the first immunotherapy to be approved for biliary tract cancer. Durvalumab monotherapy was previously approved for the treatment of advanced urothelial carcinoma but voluntarily withdrawn from the market after failing to meet its primary endpoint in follow-up studies (4).

ICIs are associated with a plethora of immune-related adverse events (irAE) caused by global immune system activation due to the inhibition of the “brakes” – PD-1/PD-L1 or CTLA-4 pathways – of the immune system (5). Immune-mediated liver injury due to ICIs (ILICI) is a subset of drug induced liver injury (DILI) resulting in indirect hepatoxicity presumably *via* intrahepatic T-cell activation (5, 6). In clinical trials, liver injury is defined based on laboratory cut-offs from the National Cancer Institute’s (NCI) Common Terminology Criteria for Adverse Events (CTCAE) (7). The degree of liver biochemistry and bilirubin elevations determine the grade of liver injury and recommendations of when to withhold or withdraw ICI therapy (8). The American Society of Clinical Oncology (ASCO) recommends evaluation for other causes of liver injury in ICI treated patients that develop elevated liver biochemistries (8). However, the incidence of ILICI compared to other causes of liver injury in patients receiving durvalumab based immunotherapy is not well defined.

Liver injury was rare in the initial clinical trials of durvalumab for NSCLC, SCLC and urothelial carcinoma, occurring in less than 1% of treated patients but elevated liver chemistries were more frequently encountered in patients with advanced biliary tract cancer (4, 9–15). Despite the low overall incidence of liver injury reported in these studies, severe or even fatal hepatic events have been reported in post-marketing surveillance (3). However, there is very limited data on the etiology of the liver injury in these trials (ILICI vs other causes) and outcome of these patients. The aim of our study was to determine the incidence, etiology, and outcomes of liver injury in 112 consecutive patients receiving durvalumab based immunotherapy at a single center who were evaluated and managed using a standardized protocol.

## Methods

### Data collection

This retrospective study was approved by the University of Michigan Medical School Institutional Review Board. All adult patients ≥ 18 years of age who received durvalumab between 1/1/2016 – 7/14/2020 were identified from the Michigan Medicine electronic medical record (EMR) using DataDirect software. Patients who were enrolled in clinical trials that of durvalumab were included in our search. Clinical characteristics including age, gender, race, ethnicity, body mass index (BMI), Eastern Cooperative Oncology Group (ECOG) performance status, and the number of durvalumab infusions received were extracted from the EMR. Medical and oncologic history was confirmed with manual chart review. Baseline laboratory data immediately before the first durvalumab infusion were also extracted, including serum aspartate aminotransferase [AST; upper limit of normal (ULN) 30 IU/L), alanine aminotransferase (ALT; ULN 35 IU/L), alkaline phosphatase (ALP; ULN 116 IU/L), total bilirubin (Tbili; ULN 1.2 mg/dL)], international normalized ratio (INR), albumin, white blood cell count, and platelets.

Additional data regarding doses, frequency, and combination durvalumab drug regimens were verified with pharmacy records. Prior chemotherapy regimens before durvalumab excluded the chemotherapy that was given with planned durvalumab consolidation therapy. Treatment regimens were defined as durvalumab “alone” if given as monotherapy, “consolidation” when given as maintenance therapy after completion of cytotoxic chemotherapy, or “combination” if durvalumab was given simultaneously with another cytotoxic agent or immunotherapy.

### Definition of liver injury

Liver injury was defined using laboratory criteria established by the Drug-Induced Liver Injury Network (DILIN) of serum ALT ≥ 5x ULN, serum ALP ≥ 2x ULN, total bilirubin ≥ 2.5 mg/dL or INR ≥ 1.5 [14]. A fold increase over baseline was used when baseline values were greater than the ULN value. Patients with isolated INR elevation on warfarin were not considered to have liver injury. In patients who met lab study criteria, serial liver biochemistries were collected to determine the course and severity of the liver injury.

### Case adjudication

Three physicians (LAS, IK, RJF) adjudicated each liver injury case and assigned a DILIN expert opinion causality score for durvalumab or any other suspect drug. This 5 point scale is defined as 1 = definite (> 95% likelihood), 2 = highly likely (75-95%), 3 = probable (50-75%), 4 = possible (25-50%), and 5 = unlikely (< 25%) (16). Patients with a DILIN causality score of 1-3 were considered to have bonafide DILI. Further review of patient data and clinical history narrowed down whether the DILI was due to durvalumab versus other drug exposures. An alternate cause was specified whenever possible for the cases assigned as a DILIN score 4 or 5. *R* ratio values were calculated at liver injury onset using the formula R = (serum ALT/ULN)/(ALP/ULN) and classified as hepatocellular (*R* > 5), mixed (*R* = 2‐5) or cholestatic (*R* < 2). Additionally, the updated Roussel Uclaf Causality Assessment Method (RUCAM) for either hepatocellular or mixed/cholestatic liver injury was calculated for each liver injury case (17). The range of RUCAM final scores is -9 to 14 with the following causality levels: ≤ excluded causality; 1–2, unlikely; 3–5, possible; 6–8, probable; and ≥ 9, highly probable (17).

### Analysis

Descriptive statistics are provided using mean (standard deviation) or median (range) for normally and non-normally distributed data, respectively. Kaplan-Meier survival curves were calculated from the time of first durvalumab infusion to death or last available follow-up in patients with and without liver injury. Between-group comparisons were performed using chi-squared and t-test. Multivariate regression was performed to better define the role of liver injury versus the presence of hepatic metastases in patient survival. Statistical significance was defined as *P* < 0.05. Analyses were completed in Microsoft Excel (Microsoft Corporation, Redmond, WA) and RStudio statistical software (Boston, MA).

## Results

### Clinical characteristics of the patient population

**Figure 1:**
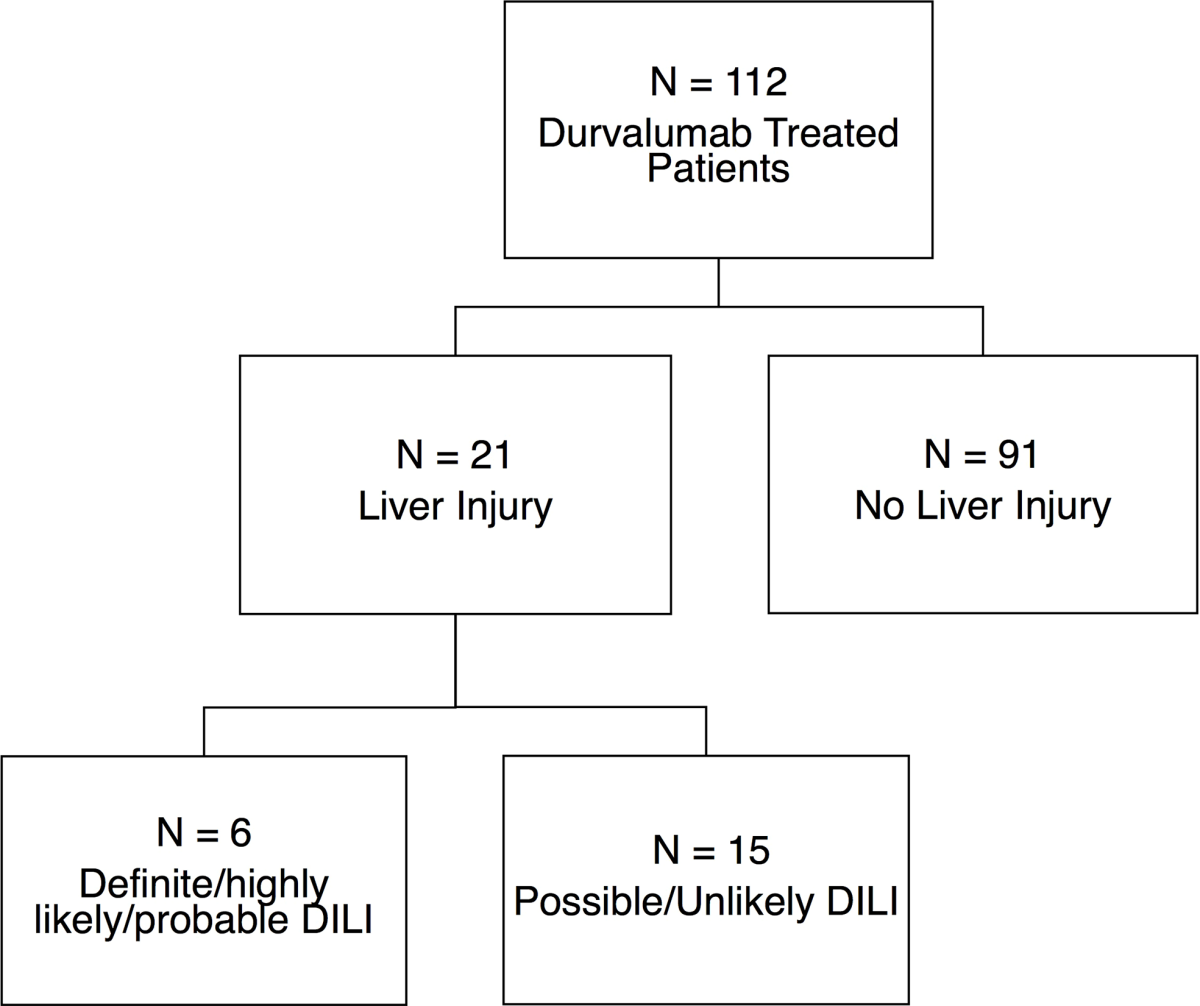
Patient population. 21 of the 112 (18.7%) patients receiving durvalumab based ICI therapy met lab criteria for liver injury while the remaining 91 (81.3%) did not. Six patients with liver injury were adjudicated as being bonafide DILI with high causality and RUCAM scores while 15 cases of liver injury were adjudicated to be due to various non-DILI causes. DILI, drug induced liver injury.

A total of 112 patients were treated with durvalumab over the study period and followed for a median of 422 days (range, 4-1463) (**Figure 1**). The median age was 65 (range, 42-85) years, 59.8% were male, 87% Caucasian, and the median ECOG Performance Status score was 1 (range, 1-3). Four of the five patients with pre-existing liver disease had prior chronic hepatitis C (HCV) infection that was successfully treated, resulting in sustained virologic response. The fifth patient had underlying non-alcoholic steatohepatitis. As expected, the median value of the baseline serum AST, ALT, ALP, and total bilirubin levels was within the normal range (**Table 1**). Of the 112 durvalumab-treated patients, 21 (19%) met laboratory criteria for liver injury after initiating therapy while 91 (81%) did not develop liver injury. Liver injury in 6 of the patients was attributed to DILI with a DILIN causality score of 1-3, while the remaining 15 liver injury cases were attributed to other etiologies.

**Table 1:** Clinical characteristics of study population.

	All Patients (N=112)	Liver Injury (n=21)	No Liver Injury (n=91)	p-value
**Age (years)**	65 [42-85]	65 [47-77]	65 [42-85]	0.618
**Male (%)**	67 (58.9)	57 (62.6)	10 (47.6)	0.206
**BMI (kg/m2)**	27.5 [18.7-44.1]	28.2 [20.7-44.1]	27.5 [18.7-40.3]	0.287
**Caucasian (%)**	97 (86.6)	18 (85.7)	79 (86.8)	0.834
**Non-Hispanic/Latino**	97 (86.6)	21 (100)	86 (94.5)	0.271
**Durvalumab Infusions**	9 [1-34]	3 [1-24]	10 [1-34]	0.018
**Cumulative Durvalumab Dose (mg)**	7500 [600-51000]	3000 [1500-36000]	9000 [600-51000]	0.076
**Duration of Follow Up (days)**	422 [4-1463]	342 [79-897]	449 [4-1463]	0.47
**ECOG (0-5)**	1 [0-3]	1 [0-1]	1 [0-3]	0.704
**Enrolled in Clinical Trial**	53 (47.3%)	16 (76.2)	37 (40.7)	0.003
**Cancer Type**				0.005
NSCLC	58 (51.8)	6 (28.6)	52 (57.1)	
SCLC	0 (0)	0 (0)	0 (0)	
Urothelial	4 (3.6)	1 (4.8)	3 (3.3)	
Other	50 (44.6)	14 (66.7)	36 (39.6)	
**Chemotherapy Regimen**				0.002
Durvalumab Alone	11 (9.8)	1 (4.8)	10 (11.0)	
Durvalumab Consolidation	59 (52.7)	5 (23.8)	54 (59.3)	
Durvalumab Combination	42 (37.5)	15 (71.4)	27 (29.7)	
**Baseline Hepatic Metastases**	26 (23.2)	11 (52.4)	15 (16.5)	< 0.001
**Prior Chemo or XRT to the Liver**	20 (37.5)	7 (33.3)	13 (14.3)	0.0454
Prior chemo	17 (15.3)	6 (28.6)	11 (12.2)	0.0613
Prior XRT	5 (4.5)	2 (9.5)	3 (3.3)	0.0164
**Comorbidities**				
Chronic kidney disease	31 (27.7)	7 (33.3)	24 (26.4)	0.521
Diabetes mellitus	25 (22.3)	7 (33.3)	18 (19.8)	0.178
Congestive heart failure	7 (6.3)	0 (0)	7 (7.7)	0.189
Liver disease	5 (4.5)	3 (14.3)	2 (2.2)	0.016
**Baseline Labs**				
AST (IU/L)	19 [10-30]	20 [14-25]	19 [10-30]	0.914
ALT (IU/L)	15 [9-31]	14 [10-25]	16 [9-31]	0.947
ALP (IU/L)	96 [60-213]	96 [60-177]	96 [63-213]	0.989
Tbili (mg/dL)	0.4 [0.2-0.9]	0.3 [0.3-0.9]	0.4 [0.2-0.9]	0.926
**Other non-hepatic irAEs**	27 (24.1)	20 (22)	7 (33.3)	0.273
**Tumor Outcome Through 7/14/2020**				0.012
Progression	43 (38.4)	14 (66.7)	29 (31.9)	
Stable/remission	67 (59.8)	7 (33.3)	60 (65.9)	
Unknown	2 (1.8)	0 (0)	2 (2.2)	
**Death**	20 (17.9)	10 (47.6)	10 (11)	< 0.001

Data presented as median [range] or n (%).

BMI, body mass index; kg/m^2^, kilogram per square meter; mg, milligram; ECOG, Eastern Cooperative Oncology Group performance status; NSCLC, non-small cell lung cancer; SCLC, small cell lung cancer; XRT, radiation therapy; AST, aspartate aminotransferase; ALT, alanine aminotransferase; ALP, alkaline phosphatase, Tbili, total bilirubin; INR, international normalized ratio; IU/L, international unit/liter; mg/dL, milligram/deciliter; irAEs, immune related adverse events.

### Liver injury and non-liver injury groups

The baseline demographics and comorbidities were similar in the 21 patients with liver injury compared to the 91 patients without liver injury (**Table 1**). However, the liver injury patients received significantly fewer durvalumab infusions (3 vs. 10, p = 0.018) compared to the patients without liver injury. The liver injury patients were also significantly more likely to have received chemotherapy or radiation therapy (XRT) to the liver in the year prior to starting durvalumab (33% vs. 14%, p = 0.045). Prior XRT to the liver was rare in both groups but more common in those with liver injury (9.5% vs. 3.3%, p = 0.0164). Although the liver injury group was more likely to have known underlying chronic liver disease (14.3% vs. 2.2%, p = 0.016) and liver metastasis (52.4% vs. 16.5%, p < 0.001), the pretreatment baseline liver biochemistries were similar in both groups.

The non-lung cancer patients enrolled in clinical trials were more likely to experience liver injury (76.2% vs. 40.7%, p = 0.003), but the time to liver injury onset was not significantly different compared to patients with lung cancer (78 vs. 181 days, p = 0.54). The types of solid organ tumors also significantly differed between the liver injury and non-liver injury groups (p = 0.005). Durvalumab given in combination with cytotoxic chemotherapy resulted in the highest rate of liver injury (71.4%% vs. 29.7%) compared to when it was given alone (4.8% vs. 11%) or as a consolidation therapy (23.8% vs. 59.3%) in both groups.

**Figure 2:**
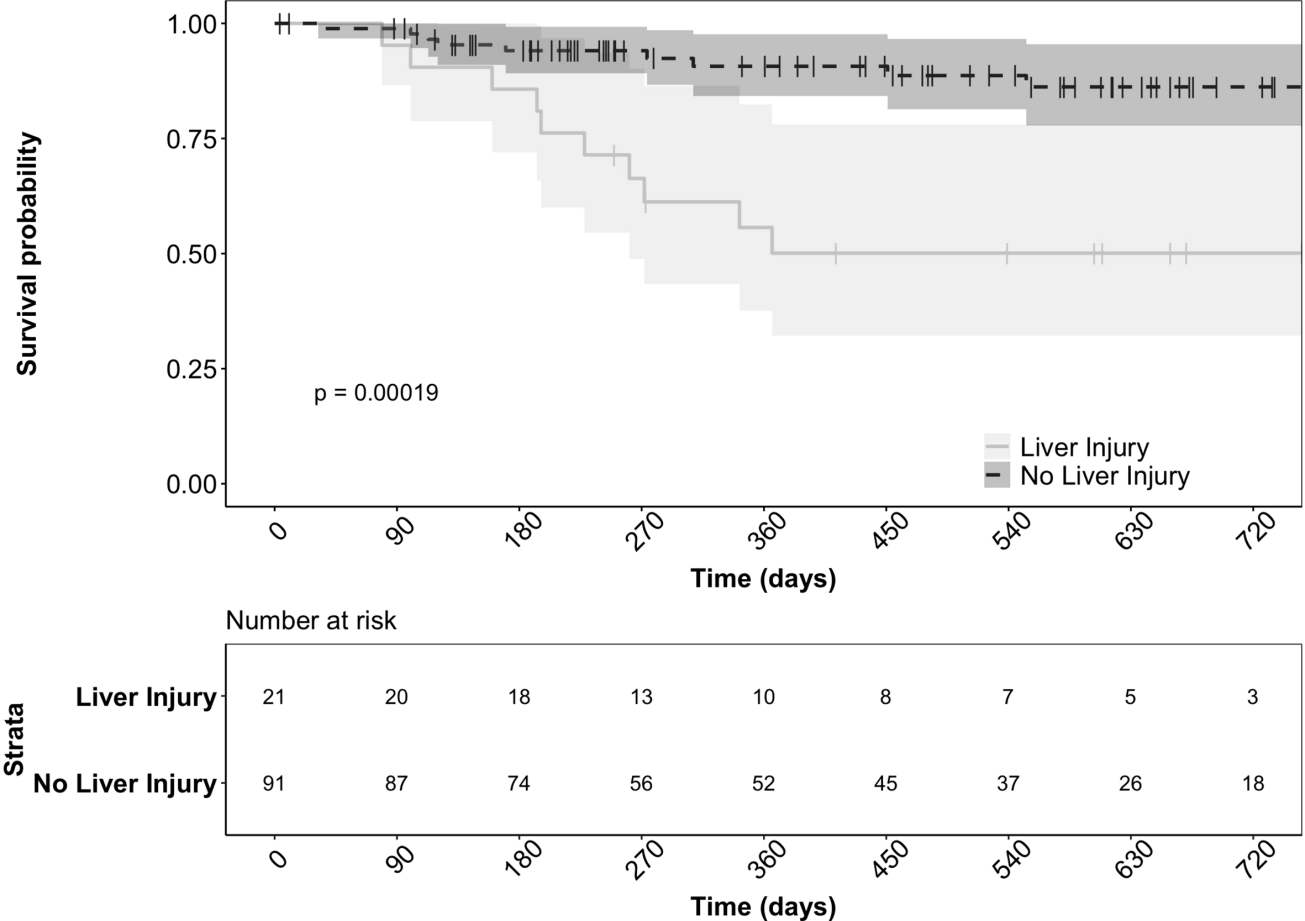
Survival of patients with and without liver injury after starting durvalumab based immunotherapy. The actuarial patient survival of the 21 patients who developed liver injury was significantly lower than those without liver injury (*p* = < 0.001 Kaplan‐Meier statistics).

There were significantly more patients with tumor progression in the liver injury group (66.6% vs. 31.9%, p = 0.011). Furthermore, the 21 liver injury patients had significantly lower survival (47.6% vs. 11%, p < 0.001) compared to the patients without liver injury (**Figure 2**). The development of liver injury during treatment and the presence of hepatic metastases at the start of therapy were both significant predictors of death in this cohort (p = 0.025 and p < 0.001, respectively) based on a multivariate regression model. However, these variables were independent without significant interaction (p = 0.172).

Other non-hepatic irAEs were noted in 27 (24%) patients, but this did not differ in the two groups.

### Causality assessment of the 21 liver injury cases

**Figure 3:**
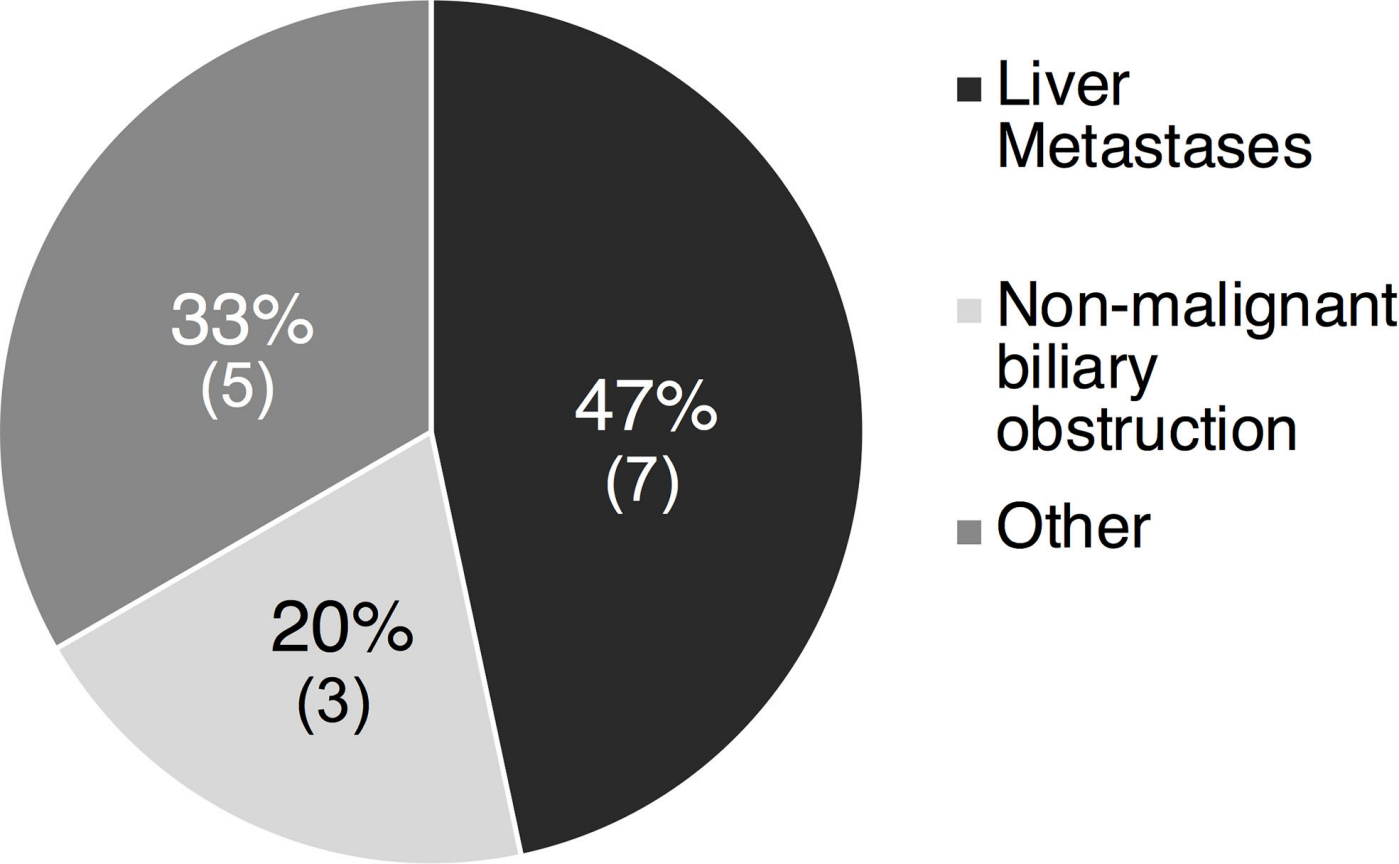
Etiology of liver injury in 15 non-DILI liver cases of the 15 patients with a causality score of 4-5 indicating possible/unlikely DILI, 7 (47%) had liver injury attributed to the presence of liver metastases and 3 (20%) due to non-malignant biliary obstruction. Other causes in 5 (33%) included Gilbert’s (1), bone metastases (1), lung abscess (1) and unknown causes (2). DILI, drug induced liver injury.

Six of the 21 (28%) liver injury cases were deemed high causality DILI cases with a DILIN expert opinion score of 1 to 3. The most common cause of non-DILI mediated liver injury was liver metastases in 7 of 15 cases (47%), followed by non-malignant biliary obstruction in 5 cases (33%) (**Figure 3**). Other less common causes of liver injury included Gilbert’s syndrome (1 case), bone metastases leading to elevated ALP (1 case), lung abscess (1 case), and unknown causes (2 cases). The RUCAM scores in these 15 patients aligned with expert opinion scores with 7 being excluded, 7 being unlikely and only 1 scored as possible.

The age, gender, race, ethnicity, and other baseline features were similar in the DILI and non-DILI groups (**Table 2**). Durvalumab exposure was also similar in the different groups with liver injury. Although the type of cancers varied between the two groups, all 6 DILI cases had been enrolled in clinical trials for experimental uses of durvalumab compared to 10 of the non-DILI cases. The median baseline AST and ALT were significantly higher in the DILI group compared to the non-DILI group. However, tumor response and patient survival were comparable between the DILI and other liver injury groups (p = 0.27) (**Supplementary Figure 1**).

**Table 2:** Clinical characteristics of the 21 patients who developed liver injury on durvalumab.

	DILI Cases (n=6)	Non-DILI Cases (n=15)	p-value
**Age (years)**	65 [47-70]	63 [49-77]	0.697
**Male (%)**	3 (50)	7 (46.7)	0.890
**BMI (kg/m2)**	32.3 [26-37]	26.1 [20.7-44.1]	0.202
**Caucasian (%)**	5 (83.3)	13 (86.7)	0.844
**Non-Hispanic/Latino(%)**	6 (100)	15 (100)	NA
**Durvalumab Infusions**	3 [1-21]	3 [1-24]	0.932
**Cumulative Durvalumab Dose (mg)**	4500 [1500-31500]	3000 [1500-36000]	0.469
**Duration of Follow Up (days)**	508 [228-756]	272 [79-897]	0.321
**ECOG (0-5)**	1 [0-1]	1 [0-1]	0.330
**Enrolled in Clinical Trial**	6 (100)	10 (66.7)	0.105
**Cancer Type**			
Colon Cancer	0 (0)	2 (13.3)	
HCC	2 (33.3)	0 (0)	
NSCLC	0 (0)	6 (40)	
Pancreatic Cancer	3 (50)	3 (20)	
RCC	1 (16.7)	3 (20)	
SCLC	0 (0)	0 (0)	
Urothelial Carcinoma	0 (0)	1 (6.7)	
**Chemo Regimen**			0.186
Durvalumab Alone	0 (0)	1 (6.7)	
Durvalumab Consolidation	0 (0)	5 (33.3)	
Durvalumab Combination	6 (100)	9 (60)	
**Baseline Hepatic Metastases (%)**	5 (83.3)	6 (40)	0.072
**Prior Chemo or XRT to the Liver**	2 (33.3)	5 (33.3)	1
Prior chemo	1 (16.7)	5 (33.3)	0.445
Prior XRT	1 (16.7)	2 (13.3)	0.844
**Liver disease**	2 (33.3)	1 (6.7)	0.115
**Baseline Labs**			
AST (IU/L)	64 [21-82]	25 [13-95]	0.022
ALT (IU/L)	54 [19-82]	22 [10-96]	0.013
ALP (IU/L)	111 [81-355]	110 [59-838]	0.766
Tbili (mg/dL)	0.7 [0.4-1]	0.4 [0.2-1.9]	0.821
**Other non-hepatic irAEs**	2 (33.3)	5 (33.3)	1
**Tumor Outcome Through 7/14/2020**			0.163
Progression	4 (50)	10 (66.7)	
Stable/remission	2 (16.7)	5 (33.3)	
Unknown	0 (0)	0 (0)	
**Death**	2 (33.3)	8 (53.3)	0.407
**Durvalumab Infusions Prior to Liver Injury**	2 [1-20]	3 [1-24]	0.742
**Days to liver injury onset**	35 [7-610]	160 [37-721]	0.519
**Labs at Liver Injury Criteria**			
AST (IU/L)	343 [36-790]	82 [14-259]	<0.001
ALT (IU/L)	415 [30-946]	96 [21-306]	0.001
ALP (IU/L)	214 [92-579]	288 [49-757]	0.636
Tbili (mg/dL)	0.9 [0.6-1.7]	0.8 [0.2-4.4]	0.403
WBC	5.1 [1.9-11.1]	8 [4.8-17.4]	0.076
% Eos	2.85 [1-6]	1.2 [1.2-10.1]	0.403
Abs Eos	100 [0-300]	100 [0-700]	0.809
***R* ratio at onset**	5.3 [0.4-17.4]	0.9 [0.2-14.5]	0.027
**Patients with LFT normalization during follow up**	6 (100)	4 (26.7)	0.002
**Time to LFT normalization (days)**	52 [14-514]	17 [2-54]	0.281
**Treated with steroids (%)**	3 (50)	0 (0)	0.003
**Durvalumab permanently discontinued**	5 (83.3)	13 (86.7)	0.843

Data presented as median [range] or n (%).

BMI, body mass index; kg/m^2^, kilogram per square meter; mg, milligram; ECOG, Eastern Cooperative Oncology Group performance status; NSCLC, non-small cell lung cancer; SCLC, small cell lung cancer; RCC, renal cell carcinoma; XRT, radiation therapy; AST, aspartate aminotransferase; ALT, alanine aminotransferase; ALP, alkaline phosphatase, Tbili, total bilirubin; INR, international normalized ratio; IU/L, international unit/liter; mg/dL, milligram/deciliter; irAEs, immune related adverse events; LFT, liver function tests.

The median time to liver injury onset was similar between the 6 DILI and 15 other liver injury cases at 35 and 160 days, respectively (p=0.519). Hepatocellular liver injury was more commonly observed in the 6 DILI patients, (50% vs. 6.7%), while cholestatic liver injury was seen more frequently in the non-DILI cases (73% vs. 33%). The DILI patients had significantly higher serum aminotransferase levels at liver injury onset (AST 343 vs. 82, p < 0.001 and ALT 415 vs. 96, p = 0.001), but there was no difference between ALP and total bilirubin levels in the two groups. All six patients with DILI had normalization of their liver biochemistries at a median of 52 days, while only 4 out of 15 (26.7%) patients with other causes of liver injury had liver enzymes normalized at a median follow-up of 17 days. As expected, the 6 DILI patients were also more likely to be treated with steroids (p = 0.003).

### Clinical features of the six DILI cases

Four of the 6 DILI cases were attributed to durvalumab, with DILIN causality scores of 1-3 for durvalumab and RUCAM scores of 6-7 indicating probable DILI (**Table 3**). Most of the DILI cases had either pancreatic adenocarcinoma or hepatocellular carcinoma. The median time to liver injury onset was 35 days (range, 7-610), following a median of 2 durvalumab infusions (range, 1-20). One patient (patient 4) developed mild jaundice during the follow-up period with a peak total bilirubin of 2.6 mg/dL. Three patients required corticosteroids for ILICI for 28-77 days. None of the six patients required the addition of mycophenolate or hospitalization due to liver injury. Durvalumab was discontinued in 2 patients (33.3%) due to liver injury, two patients due to disease progression, and one patient due to immune-mediated kidney injury. Durvalumab monotherapy was successfully reintroduced in patient six after a 4-week course of corticosteroids. Liver enzymes normalized or returned to baseline values in all six patients within a median of 52 (range, 14-514) days of follow-up. After adjudication, 2 of the 6 DILI cases were attributed to another agent, including paclitaxel in case 1 and pembrolizumab in case 5. RUCAM scores for these two DILI cases due to other drugs, the RUCAM scores for durvalumab were -2 and -4, indicating durvalumab DILI causality being excluded. In the case of pembrolizumab-associated DILI, durvalumab was discontinued due to disease progression, and the patient developed ILICI while on pembrolizumab monotherapy.

**Table 3:** Clinical characteristics of six patients who developed DILI due to any drug on durvalumab.

	All DILI cases (n=6)	Case 1	Case 2	Case 3	Case 4	Case 5	Case 6
**DILIN Score (1-5) (competing cause)**		3 (Overall - Paclitaxel) 5 for Durva	2	3	2 (Overall – Oleclumab and Durva)	3 (Overall - Pembro), 5 for Durva	2
**RUCAM Score for Durvalumab**		-2	7	6	7	-4	6
**Cancer and Stage**		Pancreatic cancer, Stage 4	HCC, Stage C	Pancreatic cancer, Stage 4	Pancreatic cancer, Stage 4	RCC, Stage 4	HCC, Stage C
**Chemo Regimen**		Durvalumab + Gemcitabine + Paclitaxel + Oleclumab	Durvalumab + tremelimumab	Durvalumab + Gemcitabine + Paclitaxel + Oleclumab	Durvalumab + Gemcitabine + Paclitaxel + Oleclumab	Durvalumab + guadecitabine; pembrolizumab after progression	Durvalumab + tremelimumab
**Onset of liver injury (days)**	35 [7-610]	42	28	10	7	100	610
**Infusions prior to liver injury**	2 [1-20]	2	1	1	1	2	20
**Time to LFT normalization (days)**	52 [14-514]	175	514	18	72	14	31
***R* ratio at onset**		0.4 (cholestatic)	17.4 (hepato-cellular)	3.2 (mixed)	7.5 (hepato-cellular)	17.3 (hepato-cellular)	1.3 (cholestatic)
**Peak Labs**							
AST (IU/L)	343 [138-790]	138	790	328	514	358	196
ALT (IU/L)	415 [138-946]	138	946	437	392	480	226
ALP (IU/L)	295 [92-586]	309	281	586	200	92	579
Tbili (mg/dL)	0.9 [0.8-2.6]	0.9	2.2	0.9	2.6	0.8	0.8
**Treated with steroids (duration)**	3 (50)	N	Y (28 days)	N	N	Y (77 days)	Y (28 days)
**Drug discontinued due to DILI**	2 (33.3)	N	Y	N	Y	N	N
**Outcome**		Drug discontinued due to tumor progression; LFTs normalized after paclitaxel discontinued due to neuropathy	Tumor progression; Drug discontinued permanently due to DILI	Stable/remission of disease; Drug discontinued due to other irEA (AKI)	Tumor progression; Drugs discontinued permanently due to DILI; LFTs normalized to baseline after discontinuation	Drug discontinued due to tumor progression; Treated with steroids for pembro induced hepatitis	Stable/remission of disease; drug restarted after treatment with steroids

DILIN, Drug-Induced Liver Injury Network; HCC, hepatocellular carcinoma; RCC, renal cell carcinoma, AST, aspartate aminotransferase; ALT, alanine aminotransferase; ALP, alkaline phosphatase, Tbili, total bilirubin; DILI, drug-induced liver injury; irAEs, immune related adverse events; LFTs, liver function tests; AKI, acute kidney injury; Y, yes; N, no, durva, durvalumab, pembro, pembrolizumab.

### Liver injury in lung cancer patients

There were 58 patients with NSCLC and none with SCLC. Six of the 58 (10.3%) developed liver injury, but the injury was attributed to non-DILI causes in all of them.

## Discussion

The number of cancer patients eligible for immunotherapy in the United States has dramatically increased from 1.5% in 2011 to an estimated 43.6% of all oncology patients in 2018, with numbers on the rise (18, 19). As of June 2022, durvalumab is currently being studied in 625 clinical trials across the globe (clinicaltrials.gov). Recent studies have shown that clinical features such as patient age are substantially higher in clinical practice compared to clinical trials, and overall survival may be diminished (19, 20). The incidence of hepatotoxicity and ILICI varies widely from 0.7% to 16% depending on the individual agent, its dose, and whether it is given in combination with an anti-CTLA4 agent (21). Therefore, there is an urgent unmet need to better understand the risk factors for developing liver injury and outcomes of patients on durvalumab therapy in clinical practice.

In this study, we describe the liver injury encountered in a diverse group of 112 cancer patients at a single center receiving durvalumab-based treatment, with nearly 50% having lung cancer and the remainder having various other solid organ tumors. Approximately 19% of these patients developed laboratory evidence of liver injury with a 5.4% incidence of DILI from any drug and 3.6% incidence of ILICI specifically attributed to durvalumab as determined by expert opinion and RUCAM scoring.

Interestingly, all cases of ILICI were in non-lung cancer patients in this cohort. A review of the published literature (**Table 4**) indicates that the overall incidence of liver injury based on the CTCAE grading system in the clinical trials of durvalumab was low, though higher in patients with biliary tract cancers (4, 9–15). The higher incidence of liver injury observed in our patients may be due, in part, to the heterogeneity of our patient population, given the inclusion of patients with HCC and preexisting liver disease who may be at increased risk of developing elevations in their liver chemistries from their underlying cancers. Given the recent approval of durvalumab in combination with chemotherapy for biliary tract cancer, it is important to understand how underlying liver and biliary tract disease may result in abnormal liver enzymes so that these patients do not have durvalumab prematurely discontinued due to presumed ILICI.

**Table 4:** Published studies of Liver Injury in patients receiving durvalumab treatment.

Publication	Study Design	Patient Population	Drug regimen	Median Duration of follow up (mon)	N	Incidence of liver injury	Definition of liver injury
**Antonia 2017^9^**	Phase III; Progression free survival	Stage III NSCLC	Durvalumab consolidation (10 mg/kg Q2W) after platinum-based chemotherapy	NR	473	0	NA
**Antonia 2018^10^**	Phase III; Overall survival	Stage III NSCLC	Durvalumab consolidation (10 mg/kg Q2W) after platinum-based chemotherapy	25.2	473	0	NA
**Antonia 2019^11^**	Phase I/II	Stage III NSCLC	Durvalumab consolidation (10 mg/kg Q2W)	27.8 – 42.5	304	2 (<1%) 1 (<1%)	Grade 1 to 4 hepatitis Grade 3 or 4 hepatitis
**Paz-Ares 2019^12^**	Phase III	Extensive stage SCLC	Durvalumab (1500 mg Q3W) + platinum–etoposide followed by durvalumab consolidation (1500 mg Q4W)	7	265	7 (2.6%) 5 (1.9%)	Any grade hepatoxicity Grade 3 or 4 hepatitis
**Goldman 2021^13^**	Phase III	Extensive stage SCLC	Durvalumab (1500 mg Q3W) + tremelimumab + platinum–etoposide followed by durvalumab consolidation (1500 mg Q4W) Vs Durvalumab (1500 mg Q3W) + platinum–etoposide followed by durvalumab consolidation (1500 mg Q4W)	25.1	531	18 (3.4%) 13 (2.4%)	Grade 1 to 4 hepatitis Grade 3 or 4 hepatitis 1 lethal case of hepatoxicity
**Powles 2017^14^**	Phase I/II	Advanced/metastatic urothelial carcinoma	Durvalumab (10 mg/kg Q2W)	NR	1012	1 (<1%) 9 (<1%)	1 lethal case of hepatoxicity Grade 3 or 4 hepatitis
**Powles 2020^4^**	Phase III	Advanced/metastatic urothelial carcinoma	Durvalumab (1500 mg Q4W) vs Durvalumab (1500 mg Q4W)+ tremelimumab followed by durvalumab (1500 mg Q4W) alone	41.2	340 345	2 (<1%) 0	2 lethal cases of hepatoxicity (grade 5 acute hepatic failure and cholestatic hepatitis)
**Oh 2022^15^**	Phase III	Advanced/metastatic biliary tract cancer	Durvalumab (1500 mg Q3W) + gemcitabine-cisplatin followed by durvalumab (1500 mg Q4W) consolidation	16.8	341	29 (8.6%) 2 (<1%)	Increased ALT Hepatic irAE grade 3/4
**durvalumab package insert^2^**		NSCLC and SCLC	Variable	NA	1889	19 (1%)	Grade 3 or 4 elevations in LFTs

NSCLC, non-small cell lung cancer; SCLC, small cell lung cancer; mg, milligrams; Mon, months; LFT, liver function tests; mg/kg, milligram/kilogram; NR, not reached; NA, No answer.

In general, the ILICI experienced by our six patients was mild, arising at a median of 35 days after the initial durvalumab infusion, and associated with favorable outcomes. Only 1 of the patients developed jaundice with a peak bilirubin of 2.6 mg/dL, and none required hospitalization or died of hepatotoxicity. Durvalumab was discontinued in two patients due to ILICI based on the recommendations for CTCAE grade 3 hepatoxicity (3, 8). Furthermore, all of the DILI patients experienced normalization of their liver biochemistries during follow-up. These data provide reassurance to clinicians with an overall favorable hepatoxicity profile of durvalumab with generally mild and reversible liver injury, whether used alone or in combination with other agents.

Development of liver injury while on durvalumab immunotherapy was associated with reduced patient survival (**Figure 2**). Recently, the presence of hepatic metastases have been shown to limit the response to ICI and result in both local and systemic T cell loss (22). Presence of hepatic metastases in comparison to other metastatic sites of disease led to diminished responses to ICI and worsened overall survival (22). Given the high prevalence of hepatic metastases in the liver injury group compared to the non-liver injury group (52.4% vs. 16.5%), we sought to determine if the if the hepatic metastases was driving the worsened survival of this group using multivariate analysis. While the presence of baseline hepatic metastases was indeed predictive of death, liver injury during treatment was independently associated with reduced survival. Further studies are needed to understand how liver injury may independently alter the effectiveness of ICI therapy as we did note increased tumor progression and decreased remission of disease in patients who developed liver injury.

Although baseline liver biochemistries were similar in patients who did and did not develop liver injury, the 6 DILI cases had significantly higher baseline AST and ALT compared to the 15 non-DILI cases (**Table 2**). Pretreatment laboratory exclusion criteria to receive ICI therapy have ranged from between > 1.5 to > 3x ULN in patients without hepatic metastases and >5x ULN for patients with hepatic metastases (5). Importantly, these patients still responded appropriately to corticosteroids, normalized their liver biochemistries, and did not have worse outcomes, suggesting that baseline liver biochemical abnormalities in these patients are not predictive of worse liver outcomes. In our study cohort, only one patient was rechallenged with durvalumab after treatment with corticosteroids.

Strengths of our study include the significant number of patients who were managed using a common clinical protocol at a single center with a median follow-up of over 1.2 years. However, a large proportion of patients were enrolled in investigational studies with heterogenous tumors and the number of ethnic minority patients was small limiting the generalizability of our findings. Nonetheless, to our knowledge, this is the largest case series describing hepatic outcomes in durvalumab treated patients in clinical practice (**Table 4**) (23, 24). Following formal causality assessment with expert opinion adjudication and confirmation with RUCAM scores, only six of the 21 liver injury cases (28%) were attributed to DILI and the remainder were attributed to disease progression or other causes (**Figure 3**). These data are similar to our recent study of pembrolizumab therapy in 420 patients with melanoma and other solid tumors (25). An additional strength of our study includes the use of the well-validated RUCAM scoring system for ICI liver toxicity (26, 27).

An important limitation of our study is the small number of verified DILI cases in this cohort of 112 patients. The retrospective nature of this study also limits our ability to establish causation. The use of EMR mining for case identification may also lead to biases and loss of interpretation of clinical context (28). Additionally, we were limited by the absence of liver biopsies and incomplete serologic evaluations to diagnose alternative etiologies of liver injury. However, recent retrospective studies have indicated that liver biopsy does not necessarily influence the outcome or management of patients with grade 3 or 4 hepatotoxicity and that corticosteroids can be initiated in patients with moderate hepatocellular injury while other causes are being investigated (29). Furthermore, the histology of ILICI has been fairly heterogeneous to date so that liver biopsy may not necessarily impact clinical management. Our data support these recommendations as patients with ILICI were able to be identified by careful review of their laboratory values, serologies, and imaging by their medical oncologist.

## Conclusion

In summary, 19% of patients receiving durvalumab-based immunotherapy developed laboratory evidence of liver injury. The liver injury was attributed to DILI in only a small proportion of these cases (26%), highlighting the need for a thorough evaluation of alternative causes of liver biochemistry elevations in oncology patients. All 4 patients with durvalumab ILICI had mild disease and recovered during follow-up. Our data and those from published clinical trials suggest that durvalumab-based immunotherapy has a favorable liver safety profile assuming that vigilant laboratory and clinical monitoring is undertaken.

## Data availability statement

The raw data supporting the conclusions of this article will be made available by the authors, without undue reservation.

## Ethics statement

The studies involving human participants were reviewed and approved by University of Michigan Institutional Review Board. Written informed consent from the participants’ legal guardian/next of kin was not required to participate in this study in accordance with the national legislation and the institutional requirements.

## Author contributions

LS, conception and design, provision of patients, collection and assembly of data, data analysis and interpretation, manuscript writing, and final approval of manuscript. IK, collection and assembly of data, data analysis and interpretation, manuscript writing, and final approval of manuscript. IT, data analysis and interpretation, and final approval of manuscript. BS, conception and design, data interpretation, and final approval of manuscript. RF, conception and design, provision of patients, data analysis and interpretation, manuscript writing, and final approval of manuscript. All authors contributed to the article and approved the submitted version.

## Conflict of interest

RF has research supported by Gilead however this is unrelated to the topic of this manuscript.

The remaining authors declare that the research was conducted in the absence of any commercial or financial relationships that could be construed as a potential conflict of interest.

## Publisher’s note

All claims expressed in this article are solely those of the authors and do not necessarily represent those of their affiliated organizations, or those of the publisher, the editors and the reviewers. Any product that may be evaluated in this article, or claim that may be made by its manufacturer, is not guaranteed or endorsed by the publisher.

## Supplementary material

Supplementary Figure 1 Survival of DILI and other liver injury patients after starting durvalumab immunotherapy.The actuarial patient survival in the six patients with DILI versus the 15 patients with non-DILI-related liver injury was similar (p = 0.27 Kaplan-Meier statistics). DILI, drug induced liver injury; DILIN, Drug Induced Liver Injury Network.
