# Supplementary figures and images for: Liver injury during durvalumab-based immunotherapy is associated with poorer patient survival: A retrospective analysis
Source: Front Oncol. 2022 Oct 24;12:984940. doi: 10.3389/fonc.2022.984940 (PMC9637844; doi:10.3389/fonc.2022.984940)

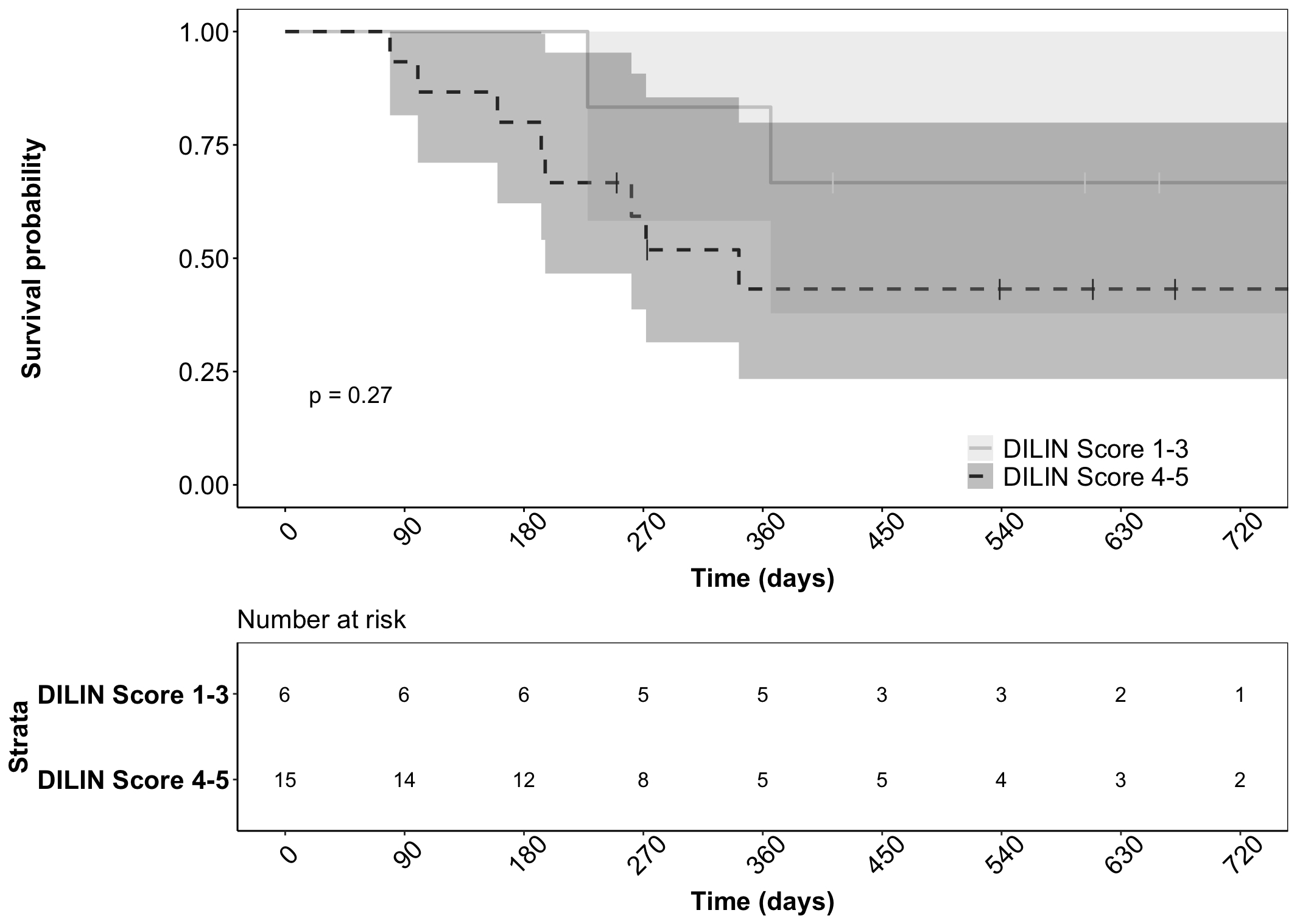

Supplement: Supplementary Figure 1 — Survival of DILI and other liver injury patients after starting durvalumab immunotherapy.The actuarial patient survival in the six patients with DILI versus the 15 patients with non-DILI-related liver injury was similar (p = 0.27 Kaplan-Meier statistics). DILI, drug induced liver injury; DILIN, Drug Induced Liver Injury Network. [file Image_1.jpeg]
